# Long‐Term Obesity Trends in Northern Sweden: Cross‐Sectional Data From the MONICA Study (1986–2022)

**DOI:** 10.1002/osp4.70170

**Published:** 2026-07-12

**Authors:** Mari Modig, Albin Dahlin Almevall, Maria Brännholm Syrjälä, Anna‐Karin Lindqvist, Maria Nordendahl, Stefan Söderberg, Viktor Oskarsson, Anna Bengtsson

**Affiliations:** ^1^ Department of Health, Education and Technology, Sunderby Research Unit – Norrbotten Luleå University of Technology Luleå Sweden; ^2^ Department of Public Health and Clinical Medicine, Research, Education and Innovation Unit – Norrbotten Umeå University Umeå Sweden; ^3^ Department of Public Health and Clinical Medicine, Björknäs Research Unit – Norrbotten Umeå University Umeå Sweden; ^4^ Department of Health, Education and Technology Luleå University of Technology Luleå Sweden; ^5^ Department of Public Health and Clinical Medicine Umeå University Umeå Sweden; ^6^ Department of Public Health and Clinical Medicine, Piteå Research Unit – Norrbotten Umeå University Umeå Sweden; ^7^ Department of Public Health and Clinical Medicine, Björkskatan Research Unit – Norrbotten Umeå University Umeå Sweden

**Keywords:** body mass index (BMI), Northern Sweden, obesity, overweight, waist circumference

## Abstract

**Background:**

Elevated BMI and central adiposity increase the risk of cardiovascular disease, type 2 diabetes, and several cancers. Obesity has risen in recent decades, with Northern Sweden exceeding the national average. Anthropometric trends in the Northern Sweden MONICA study have not been updated since 2004.

**Objective:**

To describe overweight and obesity trends in Northern Sweden from 1986 to 2022.

**Methods:**

Eight population‐based surveys were conducted between 1986 and 2022 within the Northern Sweden MONICA study. In each survey, 2000–2500 adults aged 25–74 years were invited; 12,950 participated (mean age 49.5 [SD 13.6]; 51% women), with a 68.2% response rate. Anthropometry was assessed using standardized protocols.

**Results:**

Obesity increased by 15.7 percentage points in men and 12.1 percentage points in women. By 2022, obesity had doubled since 1986, reaching 26.6% in men and 26.3% in women. Abdominal obesity also increased: with waist circumference ≥ 102 cm in men and ≥ 88 cm in women rising by 21.5 and 4.9 percentage points, respectively.

**Conclusions:**

General and abdominal obesity have substantially increased in Northern Sweden over the past four decades. These findings call for urgent, coordinated, and multi‐level interventions, as continued increases in obesity pose serious long‐term risks to population health and healthcare sustainability.

## Graphical abstract

Obesity prevalence in Northern Sweden more than doubled from 1986 to 2022, with BMI rising across all ages and sexes. Waist circumference also increased, signaling growing central adiposity and an urgent need for continued monitoring and targeted public health strategies.

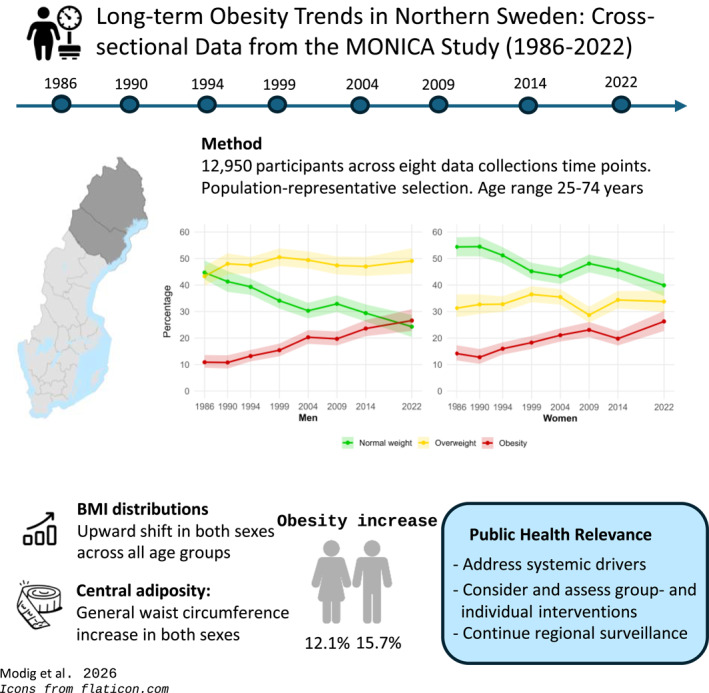

## 1 Introduction

Obesity is a global public health challenge that contributes substantially to premature mortality and chronic disease risk [1, 2]. Elevated body mass index (BMI), encompassing overweight and obesity, is accordingly associated with increased all‐cause mortality and a higher risk of cancer and cardiovascular disease, with its contribution to cardiovascular risk intensifying over recent decades [3, 4]. Obesity now affects one in eight individuals worldwide [5] and overweight and obesity together affect nearly 60% of adults in Europe [6], reflecting a sharp rise in prevalence. These trends underscore the growing burden of adiposity‐related disease and highlight the importance of sustained surveillance to identify vulnerable subgroups and inform targeted interventions for those most at risk.

In Sweden, obesity prevalence follows a global trajectory; between 2004 and 2024, the proportion of adults with overweight or obesity rose from 46% to 54% [7]. The consistent overrepresentation of men, older adults, individuals with lower educational attainment, and rural populations [8] highlights the importance of obesity surveillance beyond overall prevalence estimates.

Environmental and social determinants play key roles in shaping obesity patterns. Interactions between genetic susceptibility, sociocultural factors, and changes in food and physical activity behaviors drive BMI changes and regional variations [9]. Education and economic status influence diet and physical activity levels, and today—as food behaviors shift, recreational physical activity remains limited, and transportation demands increase—many rural areas that historically have had low obesity prevalence are facing a rise in obesity [10].

Northern Sweden provides a clear example of how these environmental and social determinants intersect. Low population density, long travel distances, and a subarctic climate with prolonged winters limit opportunities for physical activity [11, 12]. Structural inequalities, including lower educational attainment and disposable income, an older population, and reduced access to healthcare, contribute to a persistent north‐to‐south health gradient [13]. Together, these factors contribute to a more strained health situation in northern than in southern Sweden, underscoring the need for robust, region‐specific, and sustained population‐based surveys to monitor obesity patterns and disparities over time.

Against this backdrop, the Northern Sweden Monitoring of Trends and Determinants in Cardiovascular Disease (NSW‐MONICA) study offers a uniquely valuable foundation. Since 1986, it has conducted repeated population‐based surveys with standardized, objectively measured anthropometry [14], an approach matched by few international cohorts. However, published anthropometric analyses extend only through 2004 [15], leaving an 18‐year gap during a period of considerable increases in obesity prevalence.

The aim of this study was to characterize long‐term trends and contemporary prevalence of overweight and obesity in northern Sweden from 1986 to 2022. Using standardized, objectively measured anthropometric data from repeated population‐based NSW‐MONICA surveys, this study addresses a critical evidence gap and provides updated, region‐specific insights into obesity trends in Northern Sweden.

## 2 Methods

### 2.1 Design and Study Population

Within the NSW‐MONICA study, eight population‐based surveys were conducted between 1986 and 2022 in the two northernmost counties of Sweden, Norrbotten and Västerbotten, with the 2022 survey representing the most recent survey available for analysis. In each survey, 2000–2500 persons aged 25–74 years were randomly selected from population registers and stratified by sex and age group [14, 16, 17]. Persons aged 65–74 years were not included in the first two surveys in 1986 and 1990. Participation rates ranged from 81% in 1986 to 47% in 2022. Data on non‐participants between 1986 and 2009 were published previously [14], and data on non‐participants in 2022 are presented in Supporting Information S1 in Table S1. The number of participants and the rate of participation by survey year, sex, and age group are shown in Supporting Information S1 in Table S2.

The NSW‐MONICA study follows the principles of the Declaration of Helsinki [18], and the current study was reported according to the Strengthening the Reporting of Observational Studies in Epidemiology (STROBE) guidelines [19]. The NSW‐MONICA study has ethical approval from the Regional Ethical Committee at Umeå University and the Swedish Ethical Review Authority (19‐1985, 03‐375, 08‐106M, 2013/97‐31, 2021‐06369‐02). All participants provided informed and written consent.

### 2.2 Variables

Height, weight, and waist circumference were measured by trained nurses using standardized procedures. Body weight was measured to the nearest 0.2 kg (wearing light clothing and no shoes) and height to the nearest 1.0 cm. Waist circumference was measured to the nearest 0.5 cm at the midpoint between the rib margin and the iliac crest during gentle exhalation and with the feet positioned approximately 12–15 cm apart. BMI cut‐offs were based on World Health Organization (WHO) guidelines for Caucasians, where 25.0–29.9 kg/m^2^ is defined as overweight and ≥ 30.0 kg/m^2^ as obesity (Class I) [20, 21]. In a sensitivity analysis, we also looked at severe and very severe obesity (Class II: 35.0–39.9 kg/m^2^ and Class III: ≥ 40.0 kg/m^2^) [21]. Cut‐offs for waist circumference followed the National Cholesterol Education Program Expert Panel (NCEP) ATP III [22] and the International Diabetes Federation (IDF) [23] definitions of metabolic syndrome. According to the NCEP, central obesity is defined by a waist circumference ≥ 102 cm in men and ≥ 88 cm in women [22], whereas the IDF defines central obesity among persons of European descent as a waist circumference ≥ 94 cm in men and ≥ 80 cm in women [22, 23].

### 2.3 Statistical Analysis

Sex‐specific percentages and means of anthropometric measures were calculated for each survey. All estimates, except severe obesity (due to zero observations in several age groups), were either age‐standardized (according to the age distribution of the entire cohort) or given by age groups (25–34, 35–44, 45–54, 55–64, and 65–74 years). In a sensitivity analysis, the estimates were age‐standardized according to the age distribution in Norrbotten and Västerbotten in 2022 [24]. To provide a common denominator, observations with missing values in key variables (i.e., weight, BMI, or waist circumference) were excluded (*n* = 216). Pregnant women were also excluded from the analysis (0–17 individuals per survey; *n* = 69). All statistical analyses were conducted using SPSS version 29.0.1.00 and Stata version 14. Trend figures were created using R version 4.3.1, with ggplot2 used for plotting, and patchwork for figure composition. Significance was defined as a two‐tailed *p*‐value < 0.05.

## 3 Results

The study included a total of 12,950 participants; 51% were men (mean age 49.7 [SD 13.7] years) and 49% were women (mean age 49.3 [SD 13.5] years) (Supporting Information S1: Figure S1).

From 1986 to 2022, body weight, BMI, and waist circumference increased markedly in men by 9.0 kg, 1.7 kg/m^2^, and 4.6 cm, respectively (Table 1 and Supporting Information S1: Figures S2–S4). Each variable had a more modest increase between 2004 and 2022 (3.0 kg, 0.2 kg/m^2^, and 1.7 cm, respectively). Men aged 25–34 years had the greatest increase in weight (12.3 kg) and BMI (2.9 kg/m^2^) between 1986 and 2022. Waist circumference increased similarly in all age groups, exemplified by an increase of 5.9 cm among those aged 25–34 years and an increase of 5.2 cm among those aged 55–64 years (Table 1). The results were highly similar in the sensitivity analysis using a different standardization weight (Supporting Information S1: Table S3).

**TABLE 1:** Trends in weight, body mass index (BMI), and waist circumference between 1986 and 2022.

	1986	1990	1994	1999	2004	2009	2014	2022
Men (*N*)	813	755	937	889	922	847	742	444
Weight (kg)								
25–34 years	77.5 (75.9–79.1)	79.5 (77.6–81.4)	80.8 (78.9–82.7)	83.7 (81.7–85.8)	84.5 (82.4–86.6)	85.5 (83.1–88.0)	82.2 (79.3–85.2)	89.8 (85.8–93.8)
35–44 years	80.0 (78.5–81.4)	81.0 (79.4–82.6)	82.1 (80.2–84.0)	83.6 (81.8–85.4)	86.0 (83.9–88.1)	87.2 (84.8–89.6)	88.7 (85.5–91.8)	88.6 (84.8–92.5)
45–54 years	81.0 (79.3–82.8)	81.9 (80.4–83.5)	83.3 (81.6–84.9)	85.4 (83.7–87.1)	88.4 (86.4–90.4)	88.6 (86.4–90.7)	91.2 (89.0–93.4)	88.5 (85.4–91.6)
55–64 years	80.1 (78.6–81.6)	79.2 (77.7–80.7)	81.8 (80.0–83.7)	84.2 (82.5–85.9)	85.4 (83.6–87.2)	86.4 (84.6–88.1)	89.8 (87.5–92.0)	89.3 (86.3–92.3)
65–74 years	—	—	79.6 (77.9–81.3)	80.4 (78.7–82.0)	82.6 (80.9–84.4)	82.7 (81.0–84.3)	84.5 (82.5–86.5)	86.9 (84.5–89.3)
Σ 25–74 years	79.6 (78.8–80.4)	80.3 (79.4–81.1)	81.6 (80.8–82.4)	83.6 (82.8–84.4)	85.6 (84.7–86.5)	86.2 (85.3–87.2)	87.6 (86.5–88.7)	88.6 (87.2–90.1)
BMI (kg/m^2^)								
25–34 years	24.2 (23.7–24.6)	24.7 (24.2–25.2)	25.1 (24.6–25.6)	25.9 (25.3–26.4)	26.1 (25.5–26.7)	26.1 (25.5–26.8)	25.4 (24.6–26.1)	27.1 (26.0–28.2)
35–44 years	25.3 (24.8–25.7)	25.4 (25.0–25.9)	25.7 (25.1–26.2)	26.2 (25.7–26.7)	26.9 (26.3–27.4)	26.8 (26.1–27.4)	27.8 (26.9–28.6)	26.8 (25.7–27.8)
45–54 years	26.1 (25.6–26.6)	26.7 (26.3–27.1)	26.8 (26.3–27.3)	27.0 (26.5–27.5)	27.7 (27.1–28.3)	27.6 (26.9–28.2)	28.1 (27.5–28.7)	27.4 (26.4–28.3)
55–64 years	26.6 (26.1–27.0)	26.4 (25.9–26.9)	27.0 (26.4–27.5)	27.2 (26.8–27.7)	27.6 (27.0–28.1)	27.4 (26.9–28.0)	28.4 (27.7–29.0)	27.9 (27.1–28.7)
65–74 years	—	—	26.4 (25.9–26.9)	26.9 (26.4–27.4)	27.6 (27.0–28.1)	27.0 (26.5–27.5)	27.5 (26.9–28.0)	27.8 (27.1–28.5)
Σ 25–74 years	25.7 (25.5–26.0)	26.0 (25.7–26.2)	26.2 (26.0–26.5)	26.6 (26.4–26.9)	27.2 (27.0–27.5)	27.0 (26.7–27.3)	27.5 (27.2–27.8)	27.4 (27.0–27.8)
Waist (cm)								
25–34 years	88.1 (86.9–89.3)	87.4 (85.9–88.8)	88.8 (87.4–90.3)	91.7 (90.2–93.3)	91.9 (90.2–93.7)	89.7 (87.9–91.5)	86.6 (84.5–88.7)	94.0 (90.7–97.3)
35–44 years	91.9 (90.8–93.1)	90.4 (89.2–91.6)	91.5 (90.0–93.0)	93.8 (92.5–95.2)	95.2 (93.6–96.8)	93.1 (91.2–94.9)	94.4 (92.1–96.7)	94.9 (91.8–98.0)
45–54 years	94.4 (93.0–95.7)	93.6 (92.5–94.8)	95.2 (94.0–96.4)	96.2 (94.9–97.6)	98.4 (96.8–99.9)	96.5 (94.8–98.2)	97.5 (95.7–99.3)	98.6 (96.0–101.2)
55–64 years	96.7 (95.5–98.0)	93.8 (92.5–95.0)	95.9 (94.5–97.3)	97.8 (96.5–99.2)	98.3 (96.8–99.7)	97.5 (96.1–99.0)	99.6 (97.7–101.4)	101.9 (99.6–104.2)
65–74 years	—	—	95.5 (94.2–96.9)	96.7 (95.3–98.1)	98.0 (96.6–99.3)	97.2 (95.8–98.5)	98.0 (96.3–99.7)	101.2 (98.7–103.6)
Σ 25–74 years	93.6 (92.9–94.2)	92.1 (91.4–92.8)	93.5 (92.9–94.1)	95.3 (94.7–95.9)	96.5 (95.8–97.2)	94.9 (94.2–95.7)	95.5 (94.6–96.4)	98.2 (96.9–99.4)
Women (*N*)	782	785	958	919	963	855	785	554
Weight (kg)								
25–34 years	62.0 (60.6–63.5)	64.1 (62.4–65.8)	64.9 (63.3–66.6)	66.8 (64.9–68.7)	68.9 (66.7–71.1)	69.8 (67.2–72.4)	68.6 (65.9–71.4)	70.2 (66.0–74.5)
35–44 years	65.3 (63.6–67.0)	65.0 (63.5–66.4)	68.0 (66.2–69.8)	70.1 (68.1–72.0)	71.2 (69.3–73.1)	71.8 (69.5–74.1)	70.4 (68.3–72.4)	73.3 (69.9–76.6)
45–54 years	68.2 (66.5–69.9)	68.1 (66.4–69.8)	68.4 (67.1–69.7)	69.6 (67.9–71.3)	71.4 (69.5–73.3)	70.9 (69.1–72.8)	72.7 (70.7–74.8)	77.6 (74.5–80.8)
55–64 years	69.3 (67.6–71.0)	69.1 (67.5–70.8)	69.5 (67.6–71.4)	72.4 (70.7–74.1)	73.2 (71.3–75.1)	73.2 (71.2–75.3)	72.8 (70.8–74.7)	74.4 (71.9–76.9)
65–74 years	—	—	71.2 (69.2–73.3)	71.3 (69.5–73.1)	72.3 (70.4–74.1)	72.6 (70.5–74.6)	72.6 (70.3–74.8)	71.1 (68.6–73.6)
Σ 25–74 years	67.0 (66.0–67.9)	67.2 (66.3–68.1)	68.4 (67.6–69.2)	70.0 (69.1–70.8)	71.5 (70.6–72.3)	71.8 (70.8–72.7)	71.5 (70.5–72.4)	73.6 (72.2–75.1)
BMI (kg/m^2^)								
25–34 years	22.8 (22.3–23.3)	23.4 (22.8–24.0)	23.8 (23.2–24.4)	24.3 (23.7–25.0)	24.9 (24.2–25.6)	25.3 (24.3–26.2)	25.1 (24.1–26.0)	25.2 (23.7–26.6)
35–44 years	24.0 (23.5–24.6)	24.2 (23.7–24.8)	25.1 (24.5–25.8)	25.8 (25.1–26.5)	26.0 (25.3–26.7)	26.2 (25.3–27.0)	25.6 (24.9–26.3)	26.7 (25.5–27.9)
45–54 years	25.9 (25.3–26.6)	25.7 (25.1–26.3)	25.5 (25.0–26.0)	25.9 (25.4–26.5)	26.3 (25.7–27.0)	26.0 (25.4–26.7)	26.7 (26.0–27.5)	28.0 (26.9–29.1)
55–64 years	26.9 (26.2–27.5)	26.6 (25.9–27.2)	26.9 (26.2–27.6)	27.4 (26.8–28.0)	27.5 (26.9–28.2)	27.5 (26.7–28.3)	27.2 (26.5–28.0)	27.5 (26.6–28.4)
65–74 years	—	—	27.7 (26.9–28.4)	28.2 (27.5–28.9)	28.4 (27.7–29.0)	28.0 (27.2–28.8)	27.8 (27.0–28.6)	26.9 (26.0–27.8)
Σ 25–74 years	25.3 (25.0–25.7)	25.4 (25.0–25.7)	25.8 (25.5–26.1)	26.3 (26.0–26.6)	26.6 (26.3–26.9)	26.6 (26.2–26.9)	26.4 (26.1–26.8)	26.9 (26.4–27.4)
Waist (cm)								
25–34 years	80.2 (78.8–81.6)	75.9 (74.4–77.4)	78.1 (76.4–79.8)	78.6 (77.0–80.2)	80.9 (79.0–82.8)	79.2 (77.0–81.4)	77.9 (75.9–79.9)	81.1 (78.1–84.0)
35–44 years	83.1 (81.5–84.8)	77.7 (76.4–79.1)	82.3 (80.6–83.9)	83.0 (81.2–84.8)	84.5 (82.7–86.3)	82.2 (80.3–84.2)	80.9 (79.3–82.6)	85.1 (82.5–87.8)
45–54 years	86.7 (85.0–88.3)	80.6 (79.0–82.2)	83.0 (81.7–84.3)	84.6 (83.0–86.2)	86.6 (85.0–88.3)	83.9 (82.1–85.6)	85.1 (83.3–86.9)	90.5 (88.0–93.1)
55–64 years	90.6 (88.7–92.4)	83.2 (81.6–84.7)	87.7 (85.9–89.4)	88.6 (87.0–90.3)	89.9 (88.2–91.7)	88.6 (86.7–90.5)	87.7 (85.9–89.5)	91.8 (89.5–94.1)
65–74 years	—	—	89.0 (87.1–90.9)	89.4 (87.9–90.9)	90.9 (89.2–92.6)	89.1 (87.2–91.1)	88.9 (87.0–90.8)	90.8 (88.6–93.1)
Σ 25–74 years	86.4 (85.4–87.3)	80.3 (79.4–81.1)	84.0 (83.2–84.7)	84.7 (83.9–85.4)	86.7 (85.9–87.5)	84.5 (83.6–85.4)	84.0 (83.2–84.8)	87.9 (86.7–89.0)

*Note:* Values are shown as means and 95% confidence intervals, either age‐standardized (pooled analysis) or age‐specific (stratified by 10‐year age group). A total of 6349 men and 6601 women were included.

**FIGURE 1:**
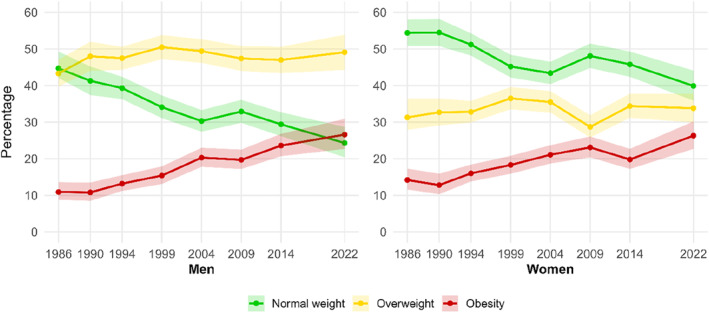
Age‐standardized trends in body mass index (BMI) between 1986 and 2022. The percentages of participants classified as normal weight (BMI < 25 kg/m^2^, green), overweight (BMI 25.0–29.9 kg/m^2^, yellow), and obesity (BMI ≥ 30.0 kg/m^2^, red) are shown as solid lines; shaded bands are 95% confidence intervals.

**FIGURE 2:**
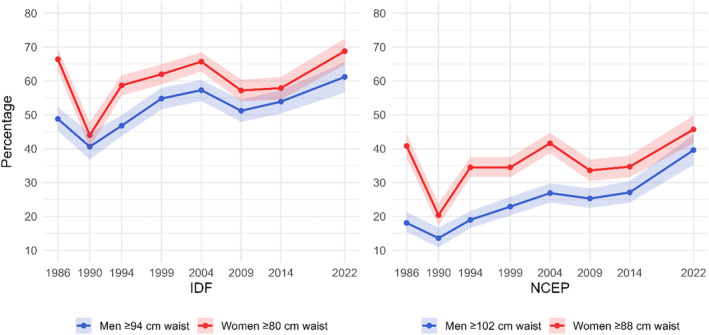
Age‐standardized trends in abdominal obesity according to the International Diabetes Federation (IDF) criteria and the National Cholesterol Education Program (NCEP) Adult Treatment Panel III (ATP III) criteria between 1986 and 2022. Solid lines represent percentages of individuals and shaded bands 95% confidence intervals.

The prevalence of overweight and obesity by survey year in men is shown in Figure 1 and Supporting Information S1: Table S4. BMI shifted from normal weight toward obesity in all age groups; specifically, the percentage of individuals in the normal weight category decreased and the percentage in the obesity category increased, but the percentage of individuals in the overweight category remained stable. A similar increase was observed for more severe obesity (Supporting Information S1: Table S5). Overall, the obesity prevalence in men increased by 15.7 percentage points, from 10.9% in 1986 to 26.6% in 2022 (Figure 1 and Supporting Information S1: Table S4). A marked increase in central obesity was observed in men between 1986 and 2022, increasing by 12.4 percentage points based on the IDF criteria (≥ 94 cm) and 21.5 percentage points based on the NCEP criteria (≥ 102 cm) (Figure 2 and Supporting Information S1: Table S6). In 2022, a total of 61.2% and 39.6% of men had abdominal obesity according to the two criteria. Supporting Information S1: Table S7 shows that height increased in men by 3.7 cm, from 175.9 cm in 1986 to 179.6 cm in 2022, whereas hip circumference (Supporting Information S1: Table S8) increased by 4.7 cm, from 98.1 to 102.8 cm.

The mean body weight, BMI, and waist circumference in women between 1986 and 2022 are also shown in Table 1. Weight increased 6.6 kg, BMI 1.6 kg/m^2^, and waist circumference 1.5 cm (Table 1, Supporting Information S1: Figures S2–S4). Similar to the male population, the increases were more modest between 2004 and 2022 (2.1 kg, 0.3 kg/m^2^, and 1.2 cm, respectively). The largest weight increase between 1986 and 2022 was observed among women aged 45–54 years (9.4 kg). The use of another standardization weight had negligible influence on the results (Supporting Information S1: Table S3).

The prevalence of overweight and obesity in women is presented in Figure 1 and Supporting Information S1 in Table S4. Between 1986 and 2022, the prevalence of obesity increased by 12.1 percentage points, from 14.2% in 1986 to 26.3% in 2022. Likewise, there was a steep increase in severe obesity (Supporting Information S1 in Table S5). The overweight category remained relatively stable with only minor fluctuations. The prevalence of central obesity also increased in women over time (Figure 2 and Supporting Information S1: Table S6); between 1986 and 2022, it increased by 2.4 percentage points according to the IDF criteria (≥ 80 cm) and 4.9 percentage points according to the NCEP criteria (≥ 88 cm). In 2022, a total of 68.8% and 45.7% of women had abdominal obesity according to the two criteria. Between 1986 and 2022, height increased in women by 2.6 cm, from 162.6 to 165.2 cm (Supporting Information S1: Table S7), whereas hip circumference increased by 4.5 cm, from 99.1 to 103.6 cm (Supporting Information S1: Table S8).

## 4 Discussion

Over a 36‐year period in Northern Sweden, obesity prevalence increased by 15.7 percentage points in men and 12.1 percentage points in women, accompanied by a decreasing proportion of individuals classified as having normal weight. Waist circumference also increased across all age groups and in both sexes, reflecting a sustained rise in central adiposity. These findings underscore a substantial and ongoing shift in body composition within this population.

The BMI trends observed in NSW‐MONICA are consistent with reports from other high‐income countries [25, 26]. The 2013 Global Burden of Disease Study showed that the rapid increases in obesity seen in developed countries since the 1980s began to level off around 2006 [27]. Similarly, our data indicate a more modest BMI increase between 2004 and 2022 compared with 1986–2004, suggesting a possible plateau. European surveillance from 2013 to 2022 ranked Sweden in the lower quartile of obesity prevalence [28], yet the considerably higher prevalence in Northern Sweden in our study (26.6% among men and 26.3% among women) highlights regional disparities requiring targeted intervention. Importantly, the continued upward trajectory in waist circumference is concerning due to its strong association with cardiometabolic morbidity [29, 30].

The current NSW‐MONICA trends also extend previous analyses from 1986 to 2004 by Lilja et al. [15]. While Lilja et al. reported a steady rise in obesity, particularly among women, with an annual BMI increase of 0.1 kg/m^2^, our updated results demonstrate that BMI increases have leveled off since 2004. Secular increases in height may partly contribute to this pattern, and although such changes can complicate BMI‐interpretation [31, 32], they are unlikely to meaningfully affect the long‐term trends observed. Importantly, both weight and waist circumference have continued to rise, reinforcing the conclusion that obesity, particularly central obesity, remains a growing public health challenge.

Disease patterns in Sweden reflect the obesity complexity. While early‐onset type 2 diabetes has increased markedly since 2006 [33], the incidence of myocardial infarction and stroke has declined [34, 35]. The declining cardiometabolic morbidity may reflect improvements in medical treatment, earlier risk detection, and reductions in other major risk factors (i.e., cigarette smoking, blood pressure, and hyperlipidemia), which could delay disease onset despite growing levels of adiposity [4, 36]. Globally, projections indicate that half the world's population will be overweight or obese by 2035 [37], further highlighting the need for improved surveillance systems.

Tolonen et al. found that responders in several population‐based studies generally had higher BMI than non‐responders [38]. However, self‐reported anthropometric data tend to underestimate BMI due to underreported weight and overreported height [31]. Based on non‐participation data in the NSW‐MONICA study from 1986 to 2009 [14] and in 2022, non‐participants reported lower BMI than measured values among participants.

Studies consistently show that self‐reported anthropometric data underestimate BMI and obesity prevalence, as individuals commonly overreport height and underreport weight [39, 40, 41]. Indeed, a systematic review from 2024 reported on underreporting of weight among women and overreporting of height among men, resulting in systematic BMI bias despite overall agreement with measured values [41]. Likewise, based on analyses from the Health Survey for England, it was reported that obesity prevalence based on self‐report was 6–7 percentage points lower than estimates derived from measured data [42]. Another review also showed consistent underestimation of overweight and obesity when using self‐reported BMI [39].

In this context, the NSW‐MONICA study's use of objectively measured weight, heigh, and waist circumference, collected by trained staff using standardized protocols across survey years, constitutes a major methodological strength, as it avoids the well‐documented reporting biases inherent in self‐reported data. Nevertheless, despite the robustness of these measured anthropometrics, the remaining limitation is the declining representativeness of the sample over time, which restricts the external comparability of our results.

Participation rates were stable during the 1980s and 1990s but declined steadily after 2004, mirroring trends in European health examination surveys, where participation fell from roughly 80% to 50%–60% by 2015 [38]. A decreasing participation rate may introduce selection bias if participation is related to body size. Based on available non‐participation data in the NSW‐MONICA cohort, individuals who declined participation generally reported lower body weight than what was measured among those who were examined. Consequently, the observed prevalence of overweight and obesity may be underestimated rather than inflated. In addition, since the overall trajectory aligns with national and international data, we consider it unlikely that selection bias explains the long‐term increase in obesity or the continued rise in waist circumference. Nonetheless, a reduction in representativeness can limit the comparability over time, especially in the youngest age groups (25–44 years), and underscores the need for altered sampling methods to maintain high participation rates.

A limitation of this study is that we were unable to report the prevalence of clinical obesity as defined by the recent Lancet Commission framework [9]. Although this multidimensional classification would have enhanced the clinical relevance of our findings, the NSW‐MONICA study lacks the broader organ system indicators required for full implementation. In addition, complete metabolic subtype data are not available for all participants as several relevant biomarkers are not available. Applying only a partial metabolic subtype would provide an incomplete and potentially misleading representation of clinical obesity. With that said, evidence from a large study of 58,053 U.S. adults by Hwang and Laiteerapong suggests that adoption of the updated diagnostic criteria may increase the prevalence compared with traditional BMI‐based definitions [43]. Future studies from Northern Sweden would benefit from incorporating the additional clinical assessments, functional measures, and comorbidity data needed to fully operationalize the clinical obesity classification and align with emerging international standards [9].

In line with the Lancet Commission's emphasis on clinical characterization, BMI and waist circumference, although valuable for monitoring population‐level trends, do not fully capture metabolic health. Differences in visceral adiposity, inflammation, insulin resistance, and muscle mass contribute to substantial heterogeneity among individuals with obesity [44, 45, 46]. These findings highlight the importance of body‐composition‐based risk assessment and support recent proposals to complement BMI with additional clinical indicators [9].

Although Sweden is not currently included in the Global Obesity Index published by Economist Impact [47], the country is clearly affected by the ongoing obesity epidemic. Being incorporated into such an index would allow Sweden to benchmark its progress, identify gaps, and evaluate its efforts in relation to other countries. Using the index framework could therefore support more systematic monitoring and strengthen Sweden's capacity to respond to rising obesity rates.

The greatest strength of the NSW‐MONICA study is its population‐based health examination design, in which trained personnel have conducted objective and uniformly standardized measurements of body weight, height, BMI, and waist circumference since the study's inception. This reliance on measured data minimizes reporting bias and ensures consistency across survey waves, enabling a reliable description of long‐term trends and contemporary obesity prevalence in Northern Sweden. This study provides updated anthropometric data from a high‐burden population, underscoring the critical importance of monitoring obesity trends to better anticipate shifts in chronic disease risk and create effective prevention strategies.

## 5 Conclusion

Over a 36‐year period in Northern Sweden, obesity prevalence more than doubled, with BMI distributions shifting upward across all age groups and in both sexes. Central adiposity also continued to increase, as reflected by increased waist circumference in both men and women. Continued monitoring and research on obesity trends will be crucial for effective public health strategies to counter obesity‐related health risks.

## Author Contributions

A.‐K.L., M.B.S., A.B., A.D.A., M.M., and S.S. contributed to the conceptualization of the study. Methodology was developed by A.‐K.L., M.B.S., A.B., A.D.A., M.M., V.O., and S.S., while formal analysis was performed by M.M., S.S., V.O., and A.D.A. Data collection was carried out by M.N. The original draft was prepared by M.M., and all authors participated in reviewing and editing the manuscript. Funding acquisition was managed by S.S., A.B., A.D.A., M.B.S., and A.‐K.L. All authors have read and approved the final version of the manuscript.

## Funding

The NSW‐MONICA study in 2022 was supported by Umeå University, the County Councils of Norrbotten and Västerbotten, the Swedish Environmental Protection Agency, and the King Gustaf V and Queen Victoria's Foundation of Freemasons. This work was supported by the Northern County Council's Regional Federation. M.M. is funded by the Unit for Research and Education, Region Norrbotten, Luleå, Sweden. The funders had no influence on the research reported in this paper.

## Conflicts of Interest

The authors declare no conflicts of interest. All authors have completed the ICMJE Conflict of Interest disclosure form, and the forms have been submitted along with the manuscript. Any potential conflicts disclosed in the forms have been addressed and are reported here.

## Supporting information


Supporting Information S1

## Data Availability

Data are available on reasonable request. Access to individual‐level data can be provided for research purposes but is restricted by laws regarding the privacy of research participants and are therefore not made publicly available. Requests for data can be sent to the Section of Biobank and Registry Support at Umeå University (contact: info.brs@umu.se).
