# Supplementary material for: Long‐Term Obesity Trends in Northern Sweden: Cross‐Sectional Data From the MONICA Study (1986–2022)
Source: Obes Sci Pract. 2026 Jul 12;12(4):e70170. doi: 10.1002/osp4.70170 (PMC13358366; doi:10.1002/osp4.70170)
Supplement: Supplementary file 1 — Supporting Information S1 [file OSP4-12-e70170-s001.docx]

Supplementary materials

**Table of contents**

[**Table S1** **Overall statistics for non-participants of NSW-MONICA in 2022** 2](#_Toc224206764)

[**Table S2 Distribution of NSW-MONICA participants by survey year, age group, and sex** 3](#_Toc224206765)

[**Figure S1 Flow chart of study participant enrollment** 4](#_Toc224206766)

[**Figure S2 Age-standardized trends in weight between 1986 and 2022 stratified by sex** 5](#_Toc224206767)

[**Figure S3 Age-standardized trends in body mass index (BMI) between 1986 and 2022 stratified by sex** 6](#_Toc224206768)

[**Figure S4 Age-standardized trends in waist circumference between 1986 and 2022 stratified by sex** 7](#_Toc224206769)

[**Table S3 Trends in** **weight, body mass index (BMI), and waist circumference between 1986 and 2022 using different standardization weights** 8](#_Toc224206770)

[**Table S4 Trends in overweight and obesity between 1986 and 2022 according to body mass index (BMI) criteria** 10](#_Toc224206771)

[**Table S5 Trends in** **severe and very severe obesity between 1986 and 2022 according to body mass index** 12](#_Toc224206772)

[**Table S6 Trends in abdominal obesity between 1986 and 2022 according to waist circumference** 13](#_Toc224206773)

[**Table S7 Trends in height between 1986 and 2022** 15](#_Toc224206774)

[**Table S8 Trends in hip circumference between 1986 and 2022** 16](#_Toc224206775)

# **Table S1** **Overall statistics for non-participants of NSW-MONICA in 2022**

|  | | Age (y) | Height (m) | Weight (kg) | BMI (kg/m^2^) |
| --- | --- | --- | --- | --- | --- |
| Men | |  |  |  |  |
|  | **Mean value** | 56.3 (SD 12.8) | 1.79 (SD 0.07) | 87.7 (SD 18.9) | 27.3 (SD 5.1) |
|  | **Difference to main cohort** | +3.0 | +/-0 | -0.7 | -0.2 |
|  | |  |  |  |  |
| Women | |  |  |  |  |
|  | **Mean** | 53.9 (SD 13.6) | 1.65 (SD 0.06) | 73.6 (SD 15.7) | 27.2 (SD 6.0) |
|  | **Difference to main cohort** | +1.5 | +/-0 | +/-0 | +0.2 |

A total of 142 respondents completed a basic questionnaire (56.8% women, 43.1% men); 75 women and 61 men had complete data on height and weight. Detailed data on non-participation in the Northern Sweden Monitoring of Trends and Determinants in Cardiovascular Disease (NSW-MONICA) study up until 2009 were published elsewhere (12). Overweight was defined as body mass index (BMI) 25.0-29.9 kg/m^2^ and obesity as BMI ≥30.0 kg/m^2^. SD, standard deviation.

# **Table S2 Distribution of NSW-MONICA participants by survey year, age group, and sex. The numbers within parenthesis (in smaller font) refer to the participation rate in each strata (expressed in %)**

|  | Age group | 1986 | 1990 | 1994 | 1999 | 2004 | 2009 | 2014 | 2022 |
| --- | --- | --- | --- | --- | --- | --- | --- | --- | --- |
| Men (N) | **25–34 y** | 172 (69) | 170 (68) | 180 (72) | 170 (68) | 161 (64) | 146 (58) | 114 (46) | 63 (25) |
|  | **35–44 y** | 205 (82) | 203 (81) | 182 (73) | 161 (64) | 169 (68) | 158 (63) | 125 (50) | 90 (36) |
|  | **45–54 y** | 225 (90) | 199 (80) | 198 (79) | 179 (72) | 182 (73) | 177 (71) | 164 (66) | 108 (43) |
|  | **55–64 y** | 212 (85) | 187 (75) | 206 (82) | 195 (78) | 213 (85) | 195 (78) | 170 (68) | 113 (45) |
|  | **65–74 y** | – | – | 174 (70) | 184 (74) | 205 (82) | 176 (70) | 173 (69) | 139 (56) |
|  |  |  |  |  |  |  |  |  |  |
| Women (N) | **25–34 y** | 179 (72) | 191 (76) | 189 (76) | 183 (73) | 177 (71) | 166 (66) | 122 (49) | 99 (40) |
|  | **35–44 y** | 202 (81) | 203 (81) | 202 (81) | 177 (71) | 198 (79) | 177 (71) | 159 (64) | 127 (51) |
|  | **45–54 y** | 209 (84) | 206 (82) | 213 (85) | 208 (83) | 198 (79) | 182 (73) | 174 (70) | 142 (57) |
|  | **55–64 y** | 199 (80) | 188 (75) | 212 (85) | 184 (74) | 206 (82) | 201 (80) | 185 (74) | 146 (58) |
|  | **65–74 y** | – | – | 163 (65) | 182 (73) | 196 (78) | 150 (60) | 158 (63) | 139 (56) |

In 1986 and 1990, 2,000 participants were invited to the Northern Sweden Monitoring of Trends and Determinants in Cardiovascular Disease (NSW-MONICA) study (age 25-64 years); from 1994 onwards, 2,500 participants were invited (age 25-74 years).

# **Figure S1 Flow chart of study participant enrollment**


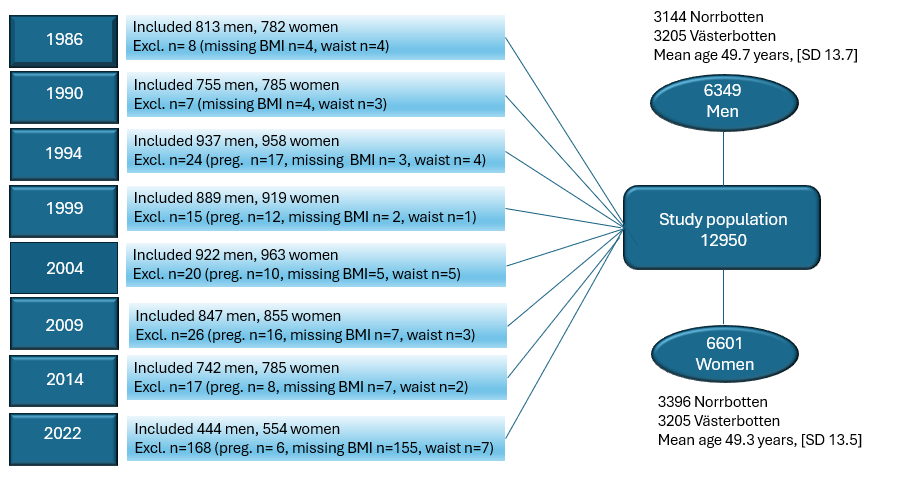


Flowchart of study population selection across survey years (1986–2022). Numbers of included participants and exclusions due to pregnancy, missing BMI, and missing waist circumference are shown. Order of exclusion: pregnancy, BMI, waist circumference. Final study population: 12,950 participants. Abbreviations: preg. = pregnant; excl. = excluded; BMI = Body Mass Index.

# **Figure S2 Age-standardized trends in weight between 1986 and 2022 stratified by sex**

**
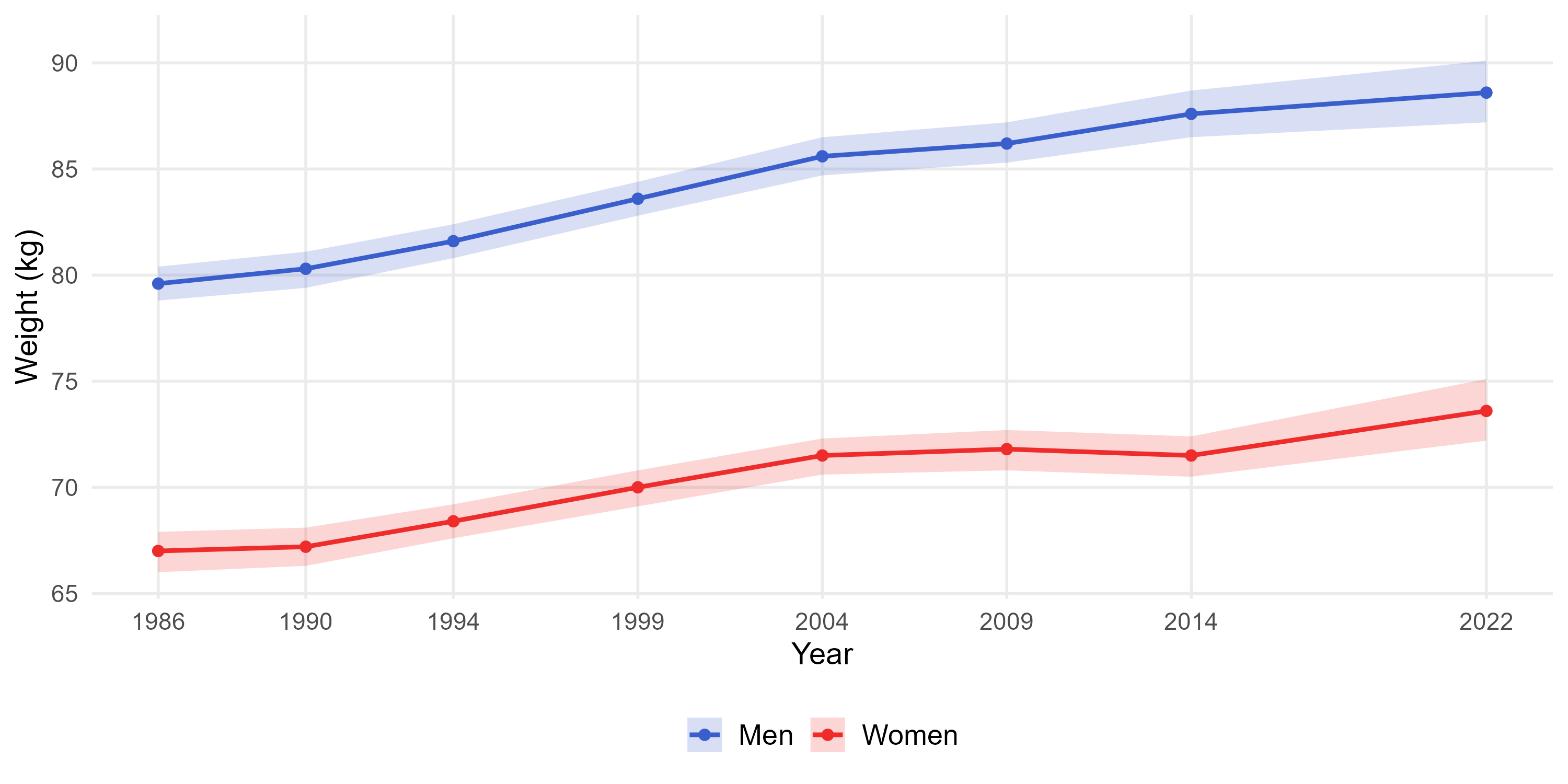
**

Solid lines represent means and shaded areas 95% confidence intervals.

# **Figure S3 Age-standardized trends in body mass index (BMI) between 1986 and 2022 stratified by sex**


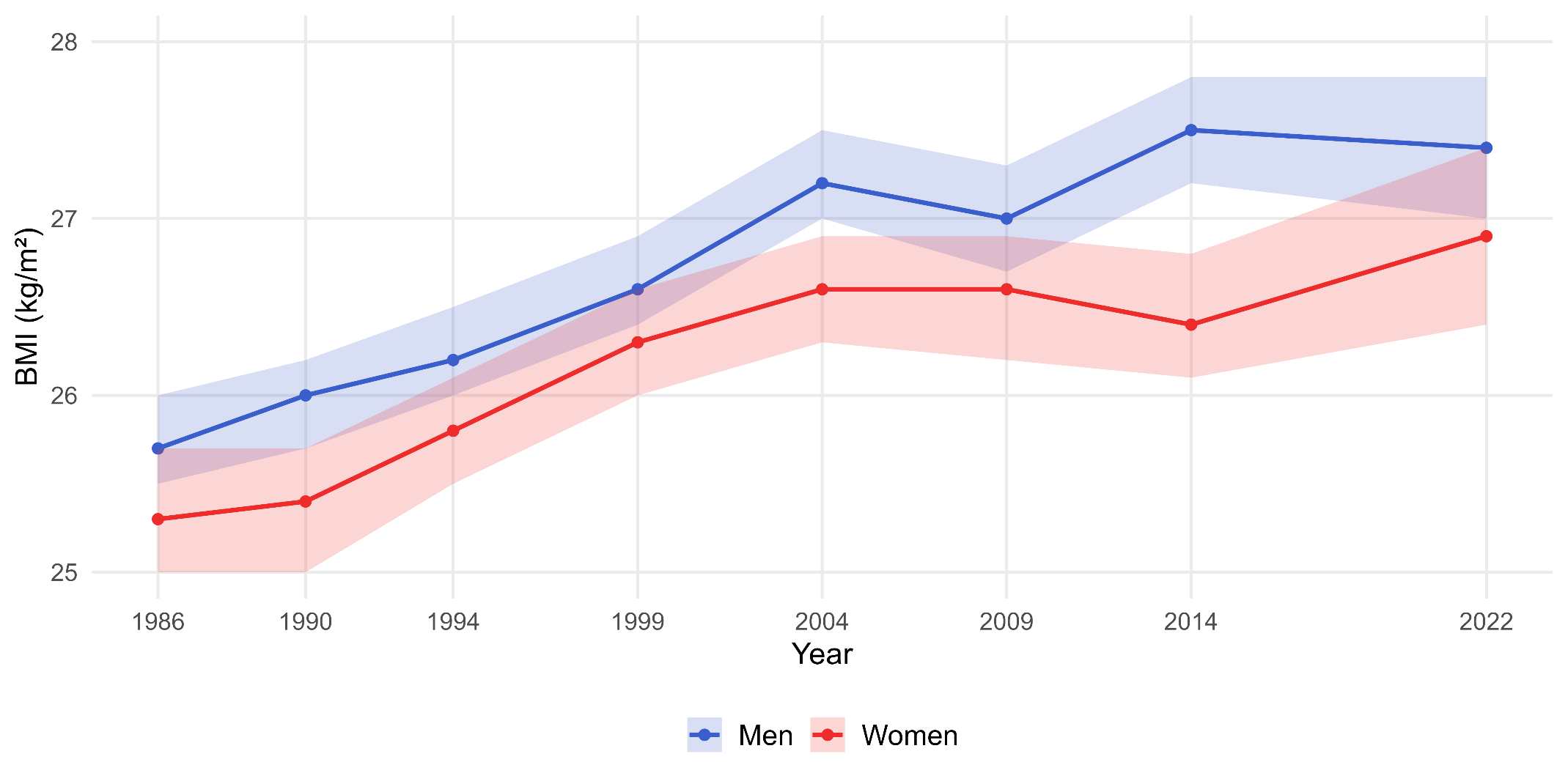


Solid lines represent means and shaded areas 95% confidence intervals.

# **Figure S4 Age-standardized trends in waist circumference between 1986 and 2022 stratified by sex**


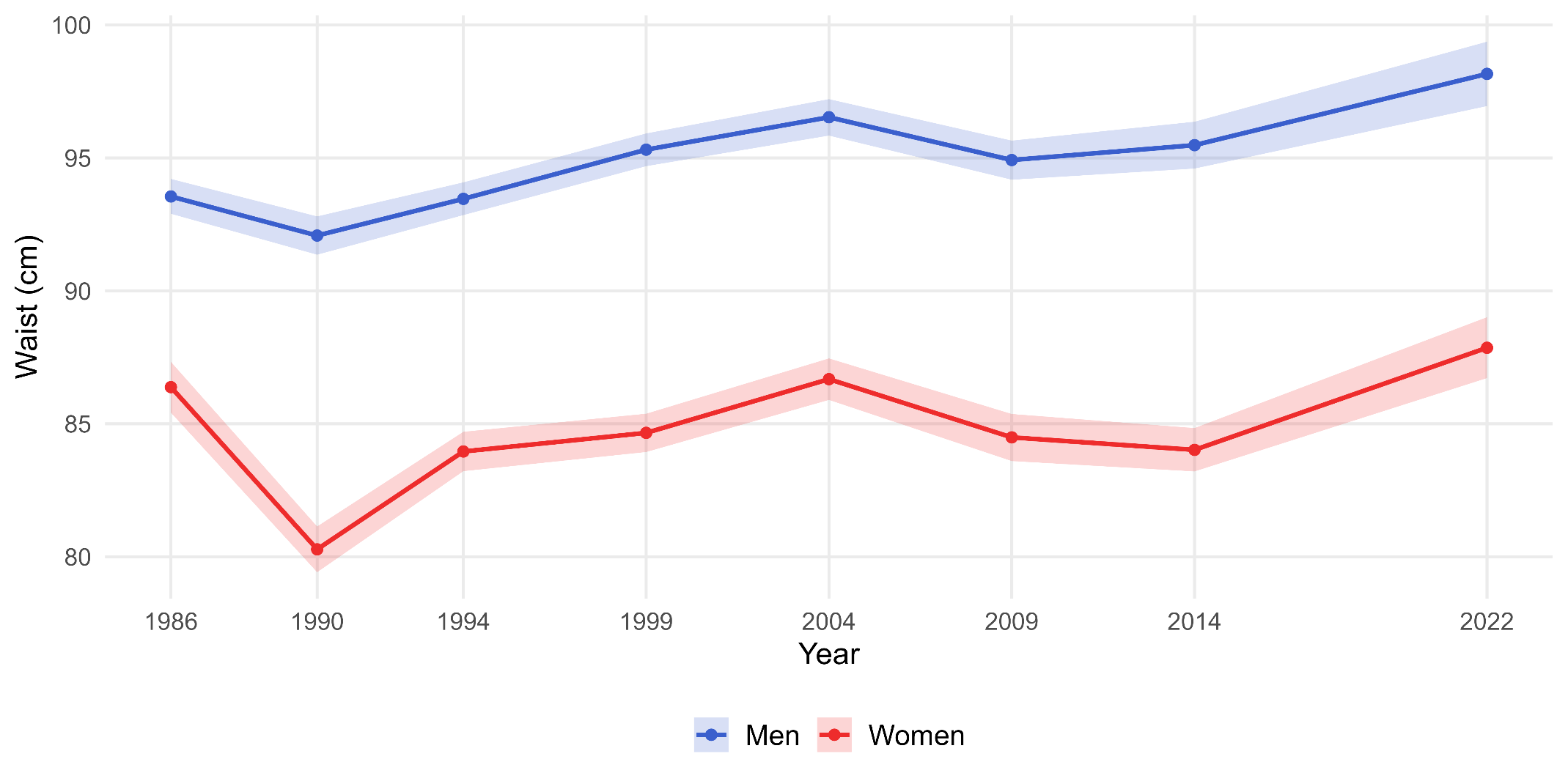


Solid lines represent means and shaded areas 95% confidence intervals.

# **Table S3 Trends in** **weight, body mass index (BMI), and waist circumference between 1986 and 2022 using different standardization weights**

|  | | | 1986 | 1990 | 1994 | 1999 | 2004 | 2009 | 2014 | 2022 |
| --- | --- | --- | --- | --- | --- | --- | --- | --- | --- | --- |
| Men (N) | | | 813 | 755 | 937 | 889 | 922 | 847 | 742 | 444 |
|  | **Weight (kg)** | |  |  |  |  |  |  |  |  |
|  | | **Age-standardized 1** | 79.6 (78.8–80.4) | 80.3 (79.4–81.1) | 81.6 (80.8–82.4) | 83.6 (82.8–84.4) | 85.6 (84.7–86.5) | 86.2 (85.3–87.2) | 87.6 (86.5–88.7) | 88.6 (87.2–90.1) |
|  | | **Age-standardized 2** | 79.5 (78.7–80.3) | 80.2 (79.4–81.1) | 81.5 (80.7–82.3) | 83.5 (82.7–84.3) | 85.5 (84.6–86.4) | 86.1 (85.2–87.1) | 87.3 (86.1–88.5) | 88.6 (87.1–90.1) |
|  | | |  |  |  |  |  |  |  |  |
|  | **BMI (kg/m^2^)*** | |  |  |  |  |  |  |  |  |
|  | | **Age-standardized 1** | 25.7 (25.5–26.0) | 26.0 (25.7–26.2) | 26.2 (26.0–26.5) | 26.6 (26.4–26.9) | 27.2 (27.0–27.5) | 27.0 (26.7–27.3) | 27.5 (27.2–27.8) | 27.4 (27.0–27.8) |
|  | | **Age-standardized 2** | 25.7 (25.4–25.9) | 25.9 (25.7–26.2) | 26.2 (25.9–26.4) | 26.6 (26.4–26.8) | 27.2 (27.0–27.5) | 27.0 (26.9–27.4) | 27.4 (27.1–27.7) | 27.3 (26.9–27.8) |
|  | | |  |  |  |  |  |  |  |  |
|  | **Waist (cm)**† | |  |  |  |  |  |  |  |  |
|  | | **Age-standardized 1** | 93.6 (92.9–94.2) | 92.1 (91.4–92.8) | 93.5 (92.9–94.1) | 95.3 (94.7–95.9) | 96.5 (95.8–97.2) | 94.9 (94.2–95.7) | 95.5 (94.6–96.4) | 98.2 (96.9–99.4) |
|  | | **Age-standardized 2** | 93.4 (92.7–94.0) | 91.9 (91.2–92.6) | 93.3 (92.6–93.9) | 95.1 (94.5–95.8) | 96.3 (95.6–97.0) | 94.7 (93.9–95.4) | 95.1 (94.2–96.0) | 97.9 (96.7–99.2) |
|  | | |  |  |  |  |  |  |  |  |
| Women (N) | | | 782 | 785 | 958 | 919 | 963 | 855 | 785 | 554 |
|  | **Weight (kg)** | |  |  |  |  |  |  |  |  |
|  | | **Age-standardized 1** | 67.0 (66.0–67.9) | 67.2 (66.3–68.1) | 68.4 (67.6–69.2) | 70.0 (69.1–70.8) | 71.5 (70.6–72.3) | 71.8 (70.8–72.7) | 71.5 (70.5–72.4) | 73.6 (72.2–75.1) |
|  | | **Age-standardized 2** | 67.1 (66.1–68.0) | 67.2 (66.2–68.2) | 68.4 (67.6–69.2) | 70.0 (69.2–70.8) | 71.4 (70.6–72.3) | 71.8 (70.8–72.8) | 71.4 (70.4–72.4) | 73.4 (72.0–74.9) |
|  | | |  |  |  |  |  |  |  |  |
|  | **BMI (kg/m^2^)*** | |  |  |  |  |  |  |  |  |
|  | | **Age-standardized 1** | 25.3 (25.0–25.7) | 25.4 (25.0–25.7) | 25.8 (25.5–26.1) | 26.3 (26.0–26.6) | 26.6 (26.3–26.9) | 26.6 (26.2–26.9) | 26.4 (26.1–26.8) | 26.9 (26.4–27.4) |
|  | | **Age-standardized 2** | 25.4 (25.0–25.7) | 25.4 (25.0–25.8) | 25.8 (25.5–26.1) | 26.3 (26.0–26.6) | 26.7 (26.4–27.0) | 26.6 (26.3–27.0) | 26.5 (26.1–26.8) | 26.9 (26.4–27.4) |
|  | | |  |  |  |  |  |  |  |  |
|  | **Waist (cm)**† | |  |  |  |  |  |  |  |  |
|  | | **Age-standardized 1** | 86.4 (85.4–87.3) | 80.3 (79.4–81.1) | 84.0 (83.2–84.7) | 84.7 (83.9–85.4) | 86.7 (85.9–87.5) | 84.5 (83.6–85.4) | 84.0 (83.2–84.8) | 87.9 (86.7–89.0) |
|  | | **Age-standardized 2** | 86.6 (85.6–87.6) | 80.3 (79.4–81.3) | 84.1 (83.3–84.8) | 84.8 (84.1–85.5) | 86.7 (85.9–87.5) | 84.7 (83.8–85.6) | 84.1 (83.3–84.9) | 87.9 (86.7–89.0) |

Values are shown as means and 95% confidence intervals, either age-standardized to age distribution of the entire study cohort (“Age-standardized 1”; main analysis) or to the age distribution of Norrbotten and Västerbotten in 2022 (“Age-standardized 2”; sensitivity analysis). A total of 6,349 men and 6,601 women were included.

*In 2022, the prevalence of obesity (BMI ≥30 kg/m^2^) using “Age-standardized 1” was 26.6% (22.7–30.9%) in men and 26.3% (22.7–30.2%) in women; using “Age-standardized 2”, it was 26.3% (22.4–30.7%) in men and 26.3% (22.7–30.2%) in women.

†In 2022, the prevalence of abdominal obesity (waist circumference ≥102 cm [men] or ≥88 cm [women]) using “Age-standardized 1” was 39.6% (35.2–44.2%) in men and 45.7% (41.6–49.8%) in women; using “Age-standardized 2”, it was 38.9% (34.5–43.5%) in men and 45.9% (41.9–50.1%) in women.

# **Table S4 Trends in overweight and obesity between 1986 and 2022 according to body mass index (BMI) criteria**

|  | |  | 1986 | | 1990 | 1994 | 1999 | 2004 | 2009 | 2014 | 2022 |
| --- | --- | --- | --- | --- | --- | --- | --- | --- | --- | --- | --- |
| Men (N) | | | | 813 | 755 | 937 | 889 | 922 | 847 | 742 | 444 |
|  | **25–34 y** | ow | 28.5 (22.2–35.7) | | 36.1 (29.2–43.6) | 35.6 (28.9–42.8) | 41.2 (34.0–48.8) | 41.9 (34.4–49.7) | 39.0 (31.4–47.2) | 37.7 (29.3–47.0) | 55.4 (42.1–67.9) |
|  | | ob | 4.1 (1.9–8.3) | | 7.1 (4.1–12.1) | 7.8 (4.7–12.7) | 11.8 (7.7–17.6) | 15.0 (10.2–21.4) | 15.8 (10.7–22.6) | 11.4 (6.7–18.7) | 19.6 (11.1–32.3) |
|  | **35–44 y** | ow | 42.4 (35.8–49.3) | | 45.8 (39.1–52.7) | 44.2 (37.1–51.5) | 47.8 (40.2–55.6) | 53.0 (45.4–60.5) | 43.0 (35.5–50.9) | 46.4 (37.8–55.2) | 43.2 (32.4–54.8) |
|  | | ob | 5.9 (3.3–10.0) | | 7.4 (4.5–11.9) | 10.5 (6.8–15.9) | 11.8 (7.6–17.8) | 16.3 (11.4–22.7) | 19.6 (14.1–26.6) | 24.0 (17.3–32.3) | 24.3 (15.8–35.5) |
|  | **45–54 y** | ow | 41.5 (35.2–48.1) | | 53.5 (46.5–60.4) | 56.6 (49.6–63.3) | 58.7 (51.3–65.7) | 55.5 (48.2–62.6) | 47.7 (40.4–55.1) | 52.1 (44.5–59.7) | 50.5 (40.4–60.6) |
|  | | ob | 13.4 (9.5–18.5) | | 16.7 (12.1–22.5) | 14.1 (9.9–19.7) | 15.1 (10.5–21.1) | 20.9 (15.6–27.4) | 25.0 (19.1–32.0) | 26.4 (20.2–33.7) | 24.7 (17.0–34.6) |
|  | **55–64 y** | ow | 51.9 (45.1–58.6) | | 49.7 (42.5–56.9) | 47.8 (41.0–54.7) | 56.4 (49.3–63.2) | 47.6 (40.9–54.4) | 53.6 (46.5–60.6) | 47.3 (39.9–54.9) | 42.3 (32.8–52.4) |
|  | | ob | 16.0 (11.7–21.6) | | 11.4 (7.5–16.8) | 20.0 (15.1–26.1) | 18.5 (13.6–24.6) | 24.3 (18.9–30.6) | 21.4 (16.1–27.7) | 30.2 (23.7–37.5) | 36.1 (27.1–46.2) |
|  | **65–74 y** | ow | ─ | | ─ | 53.2 (45.7–60.5) | 46.2 (39.1–53.5) | 48.0 (41.2–54.9) | 53.1 (45.7–60.4) | 49.7 (42.2–57.2) | 54.0 (45.2–62.6) |
|  | | ob | ─ | | ─ | 12.1 (8.0–17.9) | 20.1 (14.9–26.6) | 23.5 (18.2–29.9) | 14.9 (10.3–21.0) | 24.0 (18.1–31.0) | 28.2 (21.0–36.8) |
|  | **Σ 25–74 y** | ow | 43.3 (39.7–47.1) | | 48.0 (44.0–52.0) | 47.5 (44.3–50.6) | 50.5 (47.2–53.8) | 49.4 (46.2–52.7) | 47.4 (44.0–50.8) | 47.0 (43.4–50.6) | 49.1 (44.3–53.9) |
|  | | ob | 10.9 (8.8–13.6) | | 10.8 (8.5–13.5) | 13.2 (11.2–15.5) | 15.4 (13.1–17.9) | 20.3 (17.8–23.0) | 19.7 (17.2–22.5) | 23.6 (20.7–26.8) | 26.6 (22.7–30.9) |
|  | |  |  | |  |  |  |  |  |  |  |
| Women (N) | | | | 782 | 785 | 958 | 919 | 963 | 855 | 785 | 554 |
|  | **25–34 y** | ow | 12.4 (8.3–18.2) | | 15.9 (11.3–21.8) | 18.4 (13.3–24.9) | 24.9 (19.0–31.9) | 22.0 (16.4–28.9) | 17.4 (12.1–24.4) | 24.3 (17.3–33.1) | 18.9 (11.5–29.6) |
|  | | ob | 5.1 (2.7–9.5) | | 6.3 (3.6–10.9) | 9.2 (5.7–14.5) | 9.8 (6.2–15.3) | 14.9 (10.2–21.1) | 18.1 (12.7–25.2) | 12.2 (7.3–19.6) | 17.6 (10.4–28.1) |
|  | **35–44 y** | ow | 25.1 (19.6–31.6) | | 29.1 (23.2–35.7) | 28.9 (23.0–35.7) | 31.0 (24.6–38.3) | 35.2 (28.8–42.2) | 21.7 (16.2–28.5) | 33.5 (26.5–41.4) | 28.7 (20.7–38.3) |
|  | | ob | 8.5 (5.4–13.3) | | 5.9 (3.4–10.1) | 11.7 (7.9–17.0) | 16.7 (11.8–23.0) | 15.3 (10.9–21.1) | 22.9 (17.2–29.7) | 14.8 (10.0–21.4) | 27.7 (19.8–37.3) |
|  | **45–54 y** | ow | 31.3 (25.3–37.9) | | 30.1 (24.2–36.7) | 34.3 (28.2–40.9) | 32.4 (26.3–39.1) | 34.8 (28.5–41.8) | 33.1 (26.7–40.4) | 31.8 (25.3–39.1) | 38.2 (30.0–47.1) |
|  | | ob | 16.3 (11.9–22.0) | | 15.5 (11.2–21.2) | 11.3 (7.7–16.3) | 15.5 (11.1–21.1) | 17.2 (12.5–23.1) | 16.6 (11.8–22.7) | 24.3 (18.4–31.3) | 30.1 (22.6–38.8) |
|  | **55–64 y** | ow | 44.9 (38.1–52.0) | | 42.8 (35.9–50.0) | 40.6 (34.1–47.3) | 50.8 (43.6–58.0) | 43.4 (36.8–50.3) | 34.5 (28.2–41.4) | 39.7 (32.8–46.9) | 44.5 (36.1–53.3) |
|  | | ob | 19.7 (14.7–25.9) | | 16.6 (11.9–22.6) | 21.7 (16.6–27.8) | 23.0 (17.4–29.6) | 24.9 (19.4–31.3) | 28.5 (22.7–35.2) | 22.3 (16.8–28.9) | 27.3 (20.3–35.8) |
|  | **65–74 y** | ow | ─ | | ─ | 40.7 (33.4–48.5) | 45.6 (38.5–52.9) | 40.8 (34.1–47.9) | 38.0 (30.6–46.1) | 42.4 (34.9–50.3) | 36.7 (28.8–45.4) |
|  | | ob | ─ | | ─ | 28.4 (22.0–35.8) | 28.6 (22.5–35.6) | 34.2 (27.9–41.1) | 30.7 (23.8–38.5) | 25.9 (19.7–33.4) | 28.1 (21.0–36.6) |
|  | **Σ 25–74 y** | ow | 31.3 (27.9–35.0) | | 32.7 (29.1–36.5) | 32.8 (29.9–35.8) | 36.5 (33.5–39.6) | 35.5 (32.6–38.5) | 28.7 (25.8–31.9) | 34.4 (31.2–37.8) | 33.8 (29.9–37.8) |
|  | | ob | 14.2 (11.6–17.3) | | 12.8 (10.3–15.9) | 16.0 (13.8–18.4) | 18.3 (15.9–20.8) | 21.1 (18.7–23.7) | 23.1 (20.4–26.1) | 19.8 (17.2–22.7) | 26.3 (22.7–30.2) |

Values are shown as the percentage of individuals classified as overweight (ow; BMI 25.0–29.9 kg/m^2^) and obesity (ob; BMI ≥30.0 kg/m^2^) with 95% confidence intervals, either age-standardized (pooled analysis) or age-specific (stratified by 10-year age group).

# **Table S5 Trends in** **severe and very severe obesity between 1986 and 2022 according to body mass index**

|  | | 1986 | 1990 | 1994 | 1999 | 2004 | 2009 | 2014 | 2022 |
| --- | --- | --- | --- | --- | --- | --- | --- | --- | --- |
| Men (N) | | 813 | 755 | 937 | 889 | 922 | 847 | 742 | 444 |
| Class II Severe | 35.0–39.9 kg/m² | 1.5 (0.8–2.6) | 1.2 (0.6–2.3) | 1.8 (1.1–2.9) | 2.1 (1.4–3.3) | 2.8 (1.9–4.1 ) | 3.1 (2.1–4.5) | 5.0 (3.6–6.8) | 4.5 (2.9–6.9) |
| Class III  Very severe | ≥40.0 kg/m² | 0.1 (0.0–0.9) | No observations | 0.3 (0.1–1.0) | No observations | 1.0 (0.5–1.9) | 0.6 (0.2–1.4) | 0.5 (0.2–1.4) | 1.4 (0.6–3.0) |
|  | |  |  |  |  |  |  |  |  |
| Women (N) | | 782 | 785 | 958 | 919 | 963 | 855 | 785 | 554 |
| Class II  Severe | 35.0–39.9 kg/m² | 1.7 (1.0–2.8) | 2.3 (1.4–3.6) | 3.1 (2.2–4.4) | 3.6 (2.6–5.0) | 6.0 (4.7–7.7) | 5.5 (4.2–7.2) | 4.7 (3.4–6.4) | 7.2 (5.3–9.7) |
| Class III  Very severe | ≥40.0 kg/m² | 0.9 (0.4–1.9) | 0.9 (0.4–1.9) | 1.1 (0.6–2.1) | 1.4 (0.8–2.4) | 1.6 (0.9–2.6) | 2.5 (1.6–3.7) | 1.7 (1.0–2.8) | 3.1 (2.9–4.9) |

Values are shown as percentages and 95% confidence intervals. A total of 6,349 men and 6,601 women were included.

# **Table S6 Trends in abdominal obesity between 1986 and 2022 according to waist circumference**

|  | |  | 1986 | 1990 | 1994 | 1999 | 2004 | 2009 | 2014 | 2022 |
| --- | --- | --- | --- | --- | --- | --- | --- | --- | --- | --- |
| Men (N) | |  | 813 | 755 | 937 | 889 | 922 | 847 | 742 | 444 |
|  | **25–34 y** | ≥94 cm | 23.8 (18.0–30.8) | 21.3 (15.8–28.2) | 23.9 (18.2–30.7) | 37.6 (30.7–45.2) | 35.0 (28.0–42.7) | 30.8 (23.8–38.8) | 24.6 (17.5–33.3) | 42.9 (30.5–56.2) |
|  | | ≥102 cm | 4.7 (2.3–9.0) | 7.1 (4.1–12.1) | 8.9 (5.5–14.0) | 16.5 (11.6–22.9) | 17.5 (12.3–24.2) | 15.1 (10.1–21.9) | 9.6 (5.4–16.6) | 23.2 (13.9–36.2) |
|  | **35–44 y** | ≥94 cm | 40.0 (33.5–46.9) | 32.0 (25.9–38.8) | 39.8 (32.9–47.1) | 47.8 (40.2–55.6) | 52.4 (44.8–59.9) | 41.8 (34.3–49.6) | 44.0 (35.5–52.8) | 51.4 (40.0–62.6) |
|  | | ≥102 cm | 11.7 (8.0–16.9) | 11.8 (8.0–17.1) | 13.8 (9.5–19.7) | 19.3 (13.9–26.1) | 20.5 (15.0–27.3) | 21.5 (15.8–28.6) | 24.8 (18.0–33.2) | 32.4 (22.7–44.0) |
|  | **45–54 y** | ≥94 cm | 46.0 (39.5–52.6) | 48.0 (41.1–55.0) | 55.1 (48.0–61.9) | 59.8 (52.4–66.7) | 67.0 (59.9–73.5) | 56.8 (49.4–64.0) | 62.6 (54.9–69.7) | 64.5 (54.2–73.6) |
|  | | ≥102 cm | 22.3 (17.3–28.3) | 16.7 (12.1–22.5) | 22.2 (17.0–28.6) | 24.6 (18.8–31.4) | 29.7 (23.5–36.7) | 29.0 (22.7–36.1) | 28.2 (21.8–35.6) | 37.6 (28.3–47.9) |
|  | **55–64 y** | ≥94 cm | 65.1 (58.4–71.2) | 48.1 (41.0–55.3) | 55.6 (48.7–62.3) | 67.2 (60.3–73.4) | 64.3 (57.6–70.5) | 61.5 (54.4–68.1) | 67.5 (60.0–74.1) | 73.2 (63.5–81.1) |
|  | | ≥102 cm | 25.5 (20.0–31.8) | 14.1 (9.7–19.9) | 25.4 (19.9–31.8) | 28.7 (22.8–35.5) | 32.9 (26.8–39.5) | 29.7 (23.6–36.6) | 39.1 (32.0–46.6) | 52.6 (42.6–62.4) |
|  | **65–74 y** | ≥94 cm | – | – | 58.4 (50.9–65.5) | 59.8 (52.5–66.6) | 63.2 (56.4–69.6) | 64.0 (56.6–70.8) | 66.1 (58.6–72.8) | 73.4 (64.9–80.5) |
|  | | ≥102 cm | – | – | 23.7 (17.9–30.6) | 25.5 (19.7–32.4) | 30.9 (24.9–37.6) | 29.1 (22.9–36.3) | 31.6 (25.0–39.0) | 50.8 (42.0–59.5) |
|  | **Σ 25–74 y** | ≥94 cm | 48.8 (45.3–52.3) | 40.6 (36.8–44.6) | 46.8 (43.7–49.9) | 54.8 (51.6–58.0) | 57.3 (54.1–60.3) | 51.2 (47.9–54.5) | 53.9 (50.4–57.3) | 61.2 (56.6–65.6) |
|  | | ≥102 cm | 18.1 (15.4–21.3) | 13.6 (10.9–16.7) | 19.0 (16.6–21.6) | 22.9 (20.3–25.8) | 26.9 (24.1–29.8) | 25.3 (22.5–28.3) | 27.1 (24.1–30.4) | 39.6 (35.2–44.2) |
|  | |  |  |  |  |  |  |  |  |  |
| Women (N) | |  | 782 | 785 | 958 | 919 | 963 | 855 | 785 | 554 |
|  | **25–34 y** | ≥80 cm | 41.8 (34.7–49.2) | 21.7 (16.4–28.2) | 39.1 (32.1–46.6) | 35.8 (29.0–43.3) | 39.3 (32.2–46.9) | 36.9 (29.5–45.0) | 32.2 (24.2–41.3) | 43.2 (32.4–54.8) |
|  | | ≥88 cm | 17.5 (12.6–23.9) | 11.6 (7.8–17.1) | 17.8 (12.8–24.3) | 16.2 (11.4–22.5) | 19.6 (14.3–26.4) | 18.8 (13.3–25.9) | 13.9 (8.7–21.6) | 24.3 (15.8–35.5) |
|  | **35–44 y** | ≥80 cm | 55.3 (48.3–62.1) | 33.0 (26.9–39.8) | 52.3 (45.3–59.2) | 52.3 (44.9–59.6) | 58.2 (51.1–64.9) | 45.1 (37.9–52.6) | 47.1 (39.3–55.0) | 57.4 (47.6–66.7) |
|  | | ≥88 cm | 27.6 (21.9–34.3) | 12.3 (8.4–17.6) | 26.4 (20.7–33.0) | 25.9 (19.9–32.9) | 33.2 (26.9–40.1) | 27.4 (21.3–34.5) | 23.9 (17.8–31.2) | 38.6 (29.6–48.5) |
|  | **45–54 y** | ≥80 cm | 70.2 (63.6–76.0) | 44.7 (38.0–51.5) | 58.2 (51.5–64.7) | 62.3 (55.5–68.7) | 66.7 (59.8–72.9) | 58.6 (51.2–65.5) | 63.6 (56.1–70.4) | 81.3 (73.4–87.3) |
|  | | ≥88 cm | 40.9 (34.4–47.7) | 19.4 (14.6–25.4) | 31.5 (25.6–38.0) | 31.4 (25.4–38.1) | 44.9 (38.1–52.0) | 29.3 (23.1–36.3) | 38.7 (31.7–46.2) | 51.2 (42.4–60.0) |
|  | **55–64 y** | ≥80 cm | 81.8 (75.8–86.6) | 58.8 (51.6–65.7) | 67.9 (61.3–73.9) | 81.4 (75.1–86.4) | 80.0 (73.9–84.9) | 73.0 (66.4–78.7) | 70.7 (63.6–76.8) | 79.7 (71.8–85.8) |
|  | | ≥88 cm | 57.1 (50.1–63.8) | 29.4 (23.3–36.4) | 45.8 (39.1–52.5) | 48.1 (40.9–55.3) | 52.7 (45.8–59.5) | 47.0 (40.2–54.0) | 46.7 (39.6–54.0) | 60.2 (51.4–68.3) |
|  | **65–74 y** | ≥80 cm | – | – | 77.2 (70.0–83.0) | 81.3 (75.0–86.4) | 81.6 (75.6–86.5) | 76.7 (69.2–82.8) | 75.9 (68.6–82.0) | 82.0 (74.4–87.8) |
|  | | ≥88 cm | – | – | 53.1 (45.4–60.7) | 54.9 (47.6–62.0) | 55.1 (48.1–61.9) | 47.3 (39.4–55.4) | 51.3 (43.5–59.0) | 53.9 (45.2–62.4) |
|  | **Σ 25–74 y** | ≥80 cm | 66.4 (63.1–69.5) | 44.0 (40.3–47.8) | 58.7 (55.7–61.7) | 62.0 (59.0–64.9) | 65.7 (62.8–68.5) | 57.2 (54.0–60.4) | 57.9 (54.5–61.2) | 68.8 (64.8–72.5) |
|  | | ≥88 cm | 40.8 (37.3–44.4) | 20.4 (17.3–23.8) | 34.5 (31.7–37.5) | 34.5 (31.6–37.5) | 41.6 (38.7–44.7) | 33.6 (30.5–36.8) | 34.7 (31.6–38.0) | 45.7 (41.6–49.8) |

Values are shown as percentages of individuals and 95% confidence intervals, either age-standardized (pooled analysis) or age-specific (stratified by 10-year age group). A total of 6,349 men and 6,601 women were included. Abdominal obesity was defined by the International Diabetes Federation (IDF) cut-off (≥94 cm in men and ≥80 cm in women) or by the National Cholesterol Education Program (NCEP) Adult Treatment Panel III (ATP III) cut-off (≥102 cm in men and ≥88 cm in women).

# **Table S7 Trends in height between 1986 and 2022**

|  | | 1986 | 1990 | 1994 | 1999 | 2004 | 2009 | 2014 | 2022 |
| --- | --- | --- | --- | --- | --- | --- | --- | --- | --- |
| Men (N) | | 813 | 755 | 937 | 889 | 922 | 847 | 742 | 444 |
|  | **Height (cm)** | 175.9 | 175.8 | 176.3 | 177.0 | 177.3 | 178.6 | 178.3 | 179.6 |
|  | **95% CI** | 175.5–176.4 | 175.3–176.3 | 175.9–176.8 | 176.6–177.5 | 176.9–177.7 | 178.2–179.1 | 177.8–178.8 | 178.9–180.2 |
|  | |  |  |  |  |  |  |  |  |
| Women (N) | | 782 | 785 | 958 | 919 | 963 | 855 | 785 | 554 |
|  | **Height (cm)** | 162.6 | 162.9 | 162.9 | 163.2 | 163.9 | 164.5 | 164.4 | 165.2 |
|  | **95% CI** | 162.2–163.1 | 162.4–163.3 | 162.6–163.3 | 162.9–163.6 | 163.5–164.3 | 164.1–164.9 | 164.0–164.9 | 164.7–165.8 |

Values are shown as age-standardized means and 95% confidence intervals (CIs).

# **Table S8 Trends in hip circumference between 1986 and 2022**

|  | | 1986 | 1990 | 1994 | 1999 | 2004 | 2009 | 2014 | 2022 |
| --- | --- | --- | --- | --- | --- | --- | --- | --- | --- |
| Men (N) | | 813 | 755 | 935 | 889 | 920 | 812 | 741 | 442 |
|  | **Hip (cm)** | 98.1 | 98.0 | 99.5 | 103.1 | 100.6 | 100.7 | 98.2 | 102.8 |
|  | **95% CI** | 97.7–98.5 | 97.6–98.5 | 99.1–100.0 | 102.6–103.5 | 100.2–101.1 | 100.2–101.2 | 97.7–98.8 | 102.0–103.5 |
|  | |  |  |  |  |  |  |  |  |
| Women (N) | | 782 | 785 | 957 | 918 | 963 | 855 | 782 | 554 |
|  | **Hip (cm)** | 99.1 | 98.7 | 100.7 | 103.4 | 101.5 | 101.8 | 98.8 | 103.6 |
|  | **95% CI** | 98.5–99.8 | 98.0–99.4 | 100.1–101.2 | 102.9–104.0 | 100.9–102.1 | 101.1–102.5 | 98.2–99.5 | 102.5–104.6 |

Values are shown as age-standardized means and 95% confidence intervals (CIs). Five women and 42 men had missing values for hip circumference.
